# Skeletal muscle alkaline Pi pool is decreased in overweight-to-obese sedentary subjects and relates to mitochondrial capacity and phosphodiester content

**DOI:** 10.1038/srep20087

**Published:** 2016-02-03

**Authors:** Ladislav Valkovič, Marek Chmelík, Barbara Ukropcová, Thomas Heckmann, Wolfgang Bogner, Ivan Frollo, Harald Tschan, Michael Krebs, Norbert Bachl, Jozef Ukropec, Siegfried Trattnig, Martin Krššák

**Affiliations:** 1 High Field MR Centre, Department of Biomedical Imaging and Image-guided Therapy, Medical University of Vienna, Vienna, Austria; 2 Christian Doppler Laboratory for Clinical Molecular MR Imaging, Vienna, Austria; 3 Department of Imaging Methods, Institute of Measurement Science, Slovak Academy of Sciences, Bratislava, Slovakia; 4 Oxford Centre for Clinical MR Research (OCMR), University of Oxford, Oxford, United Kingdom; 5 Obesity section, Diabetes and Metabolic Disease Laboratory, Institute of Experimental Endocrinology, Slovak Academy of Sciences, Bratislava, Slovakia; 6 Institute of Pathophysiology, Faculty of Medicine, Comenius University, Bratislava, Slovakia; 7 Department of Sports and Physiological Performance, Centre of Sports Science, University of Vienna, Vienna, Austria; 8 Division of Endocrinology and Metabolism, Department of Internal Medicine III, Medical University of Vienna, Vienna, Austria

## Abstract

Defects in skeletal muscle energy metabolism are indicative of systemic disorders such as obesity or type 2 diabetes. Phosphorus magnetic resonance spectroscopy (^31^P-MRS), in particularly dynamic ^31^P-MRS, provides a powerful tool for the non-invasive investigation of muscular oxidative metabolism. The increase in spectral and temporal resolution of ^31^P-MRS at ultra high fields (i.e., 7T) uncovers new potential for previously implemented techniques, e.g., saturation transfer (ST) or highly resolved static spectra. In this study, we aimed to investigate the differences in muscle metabolism between overweight-to-obese sedentary (Ob/Sed) and lean active (L/Ac) individuals through dynamic, static, and ST ^31^P-MRS at 7T. In addition, as the dynamic ^31^P-MRS requires a complex setup and patient exercise, our aim was to identify an alternative technique that might provide a biomarker of oxidative metabolism. The Ob/Sed group exhibited lower mitochondrial capacity, and, in addition, static ^31^P-MRS also revealed differences in the Pi-to-ATP exchange flux, the alkaline Pi-pool, and glycero-phosphocholine concentrations between the groups. In addition to these differences, we have identified correlations between dynamically measured oxidative flux and static concentrations of the alkaline Pi-pool and glycero-phosphocholine, suggesting the possibility of using high spectral resolution ^31^P-MRS data, acquired at rest, as a marker of oxidative metabolism.

Obesity, resulting from an imbalance between energy intake and expenditure, is a worldwide epidemic associated with insulin resistance syndrome. Given that skeletal muscle accounts for almost half the total body mass and is responsible for the majority of glucose uptake and glycogen storage in response to insulin stimulus^1^, the investigation of muscle energy expenditure is of particular importance with regard to the pathogenesis of obesity and metabolic syndrome. Recent studies showed that insulin resistance relates to abnormalities in energy metabolism, not only of skeletal muscle^2^,^3^,^4^, but also of the heart^5^ and liver^6^. The contractile activity of skeletal muscle is primarily regulated by the ATP synthesis rate^7^, which, under aerobic conditions in exercised muscle, is determined mainly by the oxidative phosphorylation capacity of mitochondria. Changes in muscle energy metabolism related to mitochondrial dysfunction could indicate defects in lipid metabolism (i.e., fatty acid oxidation)^8^, potentially resulting in the progression of metabolic disease, such as type 2 diabetes, even in a young, overweight-to-obese, sedentary population^9^,^10^,^11^.

The non-invasive detection of intramyocellular energy metabolites (i.e., phospocreatine [PCr], ATP, and inorganic phosphate [Pi]) is possible through phosphorous magnetic resonance spectroscopy (^31^P-MRS), which provides an ideal tool for the *in vivo* monitoring of cellular energy status and metabolism^7^,^12^. Dynamic ^31^P-MRS, during exercise and recovery, in particular, allows direct estimation of the oxidative ATP synthesis rate in challenged muscle^12^,^13^,^14^,^15^, which reflects maximal mitochondrial capacity^7^. Altered mitochondrial metabolismis associated with obesity, elevated fasting glucose or insulin resistance^16^,^17^,^18^,^19^,^20^. As the dynamic examinations require a complex setup, e.g., dedicated ergometers, and patient compliance throughout the whole exercise protocol, an alternative ^31^P-MRS technique for the assessment of energy metabolism at rest would constitute a significant advantage. The measurement of resting Pi-to-ATP flux (F_ATP_) using ^31^P-MRS saturation transfer (ST), correlates with the findings of dynamic experiments^21^,^22^. Although the absolute values of F_ATP_ do not provide a direct measure of oxidative metabolism^23^, it has also been related to insulin resistance^24^,^25^.

Recently, the use of ^31^P-MR spectra, measured in the equilibrium state, has been promoted to obtain similar information about muscle energy metabolism. In particular, the concentration of phosphodiesters ([PDE]) was shown to correlate with the Pi-to-ATP flux^26^. Moreover, an alkaline Pi pool (Pi_2_) has been detected *in vivo* at ultra-high field (i.e., 7T)^27^, and related to the PCr re-synthesis rate after exercise^28^.

Our aim was to compare the skeletal muscle metabolism of overweight-to-obese sedentary (Ob/Sed) subjects, who are prone to type 2 diabetes, and lean active (L/Ac) individuals, using static and dynamic ^31^P-MRS measurements in the quadriceps femoris muscle at 7 T. In addition, the interrelations between the derived parameters were investigated to determine possible alternatives to exercise-recovery experiments.

## Results

### Between groups comparison

**Figure 1:**
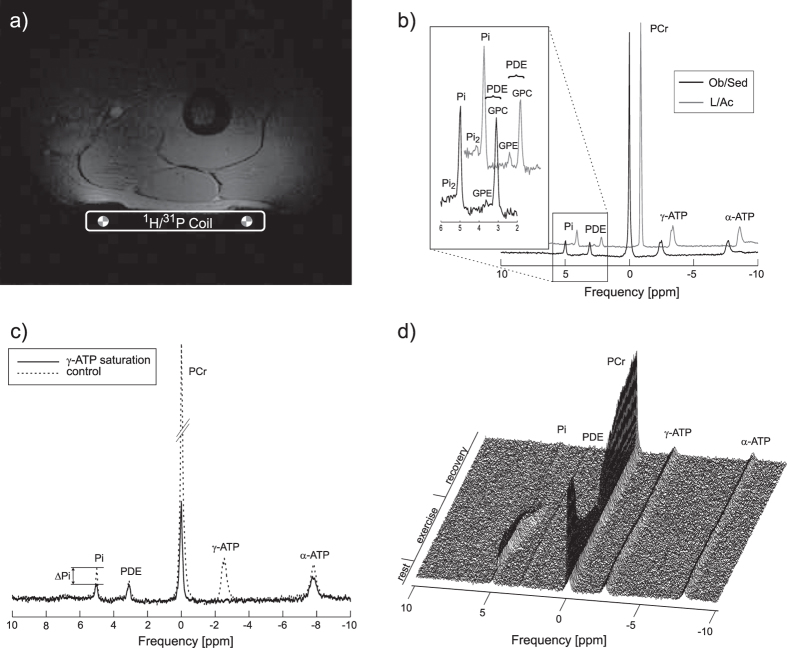
(**a**) An *in vivo* localizer image of the human thigh with the depicted coil position. (**b**) Highly spectrally resolved representative ^31^P-MR spectra from an obese sedentary and lean active subject, scaled to PCr signal intensity. The area of Pi and PDE peaks is enlarged. Note higher Pi_2_ and lower PDE signal intensity in the L/Ac subject. (**c**) Saturation transfer spectra showing the effect of γ-ATP saturation (solid line) on its chemical exchange partner, Pi, compared with the control experiment (dashed line). (**d**) Time course of the ^31^P spectra during a dynamic ^31^P-MRS experiment. Note the PCr signal depletion during exercise and its re-synthesis during recovery.

**Figure 2:**
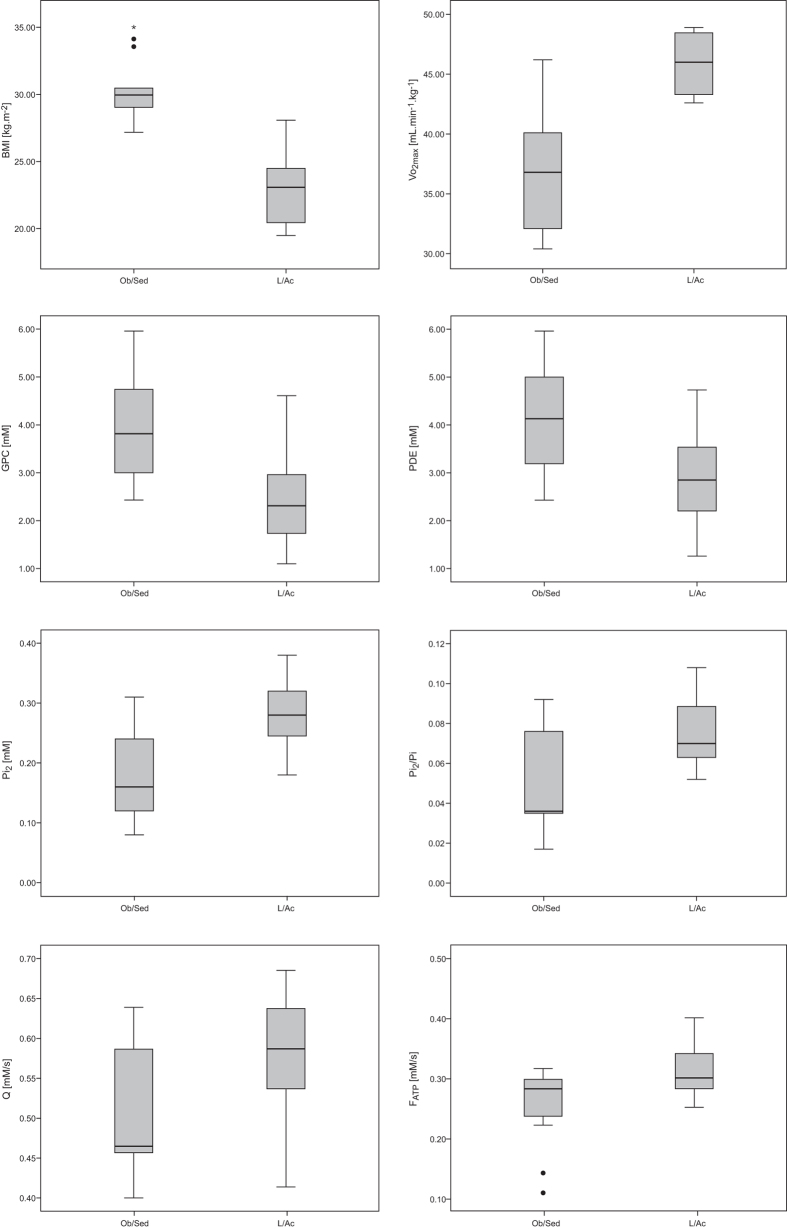
Box plots depicting the significantly different physiological and metabolic parameters between the two groups. The solid lines represent the median, boxes represent lower and upper quartiles, and whiskers the minimum and maximum. Outliers and extreme outliers are denoted by circles and stars, respectively. The outliers were also taken into account for all statistical tests.

In addition to a significantly higher BMI and lower VO_2max_, the Ob/Sed individuals also differed from the L/Ac volunteers in the metabolic parameters derived from ^31^P-MRS. The concentration of the main muscular PDE (i.e., glycero-phosphocholine [GPC]), as well as the total [PDE], were significantly higher, while the concentration of the alkaline Pi-pool ([Pi_2_]) and its ratio to the main Pi concentration ([Pi_1_]), i.e., (Pi_2_/Pi), were significantly lower in the Ob/Sed group compared to the L/Ac group. In addition, the group of Ob/Sed subjects had significantly lower mitochondrial capacity (Q_max_) and Pi-to-ATP exchange flux (F_ATP_) values compared to the L/Ac group. Detailed information about the measured physiological and muscle energy metabolism parameters are listed in Table 1. In Fig. 1 are depicted representative ^31^P-MR spectra acquired at rest and during the exercise-recovery experiment and Fig. 2 depicts the comparison between the groups.

### Correlations between the measured parameters

The measured concentration of PDE in the quadriceps muscle correlated positively with both age (r = 0.45, p = 0.014) and BMI (r = 0.62, p = 0.0004). BMI correlated negatively with the [Pi_2_] (r = −0.56, p = 0.002), as well as with the Pi_2_/Pi ratio (r = −0.44, p = 0.023) and Q_max_ (r = −0.39, p = 0.039). The calculated F_ATP_ was also found to be negatively correlated with age (r = −0.48, p = 0.009) and BMI (r = −0.51, p = 0.007).

**Figure 3:**
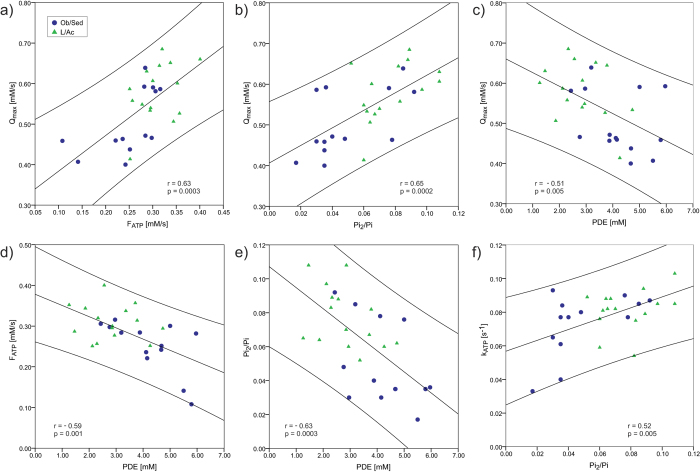
Plots of correlations between myocellular energy metabolism parameters measured by dynamic and static ^31^P-MRS in Ob/Sed (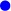) andL/Ac (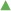) individuals: (**a**) mitochondrial capacity (Q_max_) with the Pi-to-ATP forward metabolic flux at rest (F_ATP_); (**b**) Q_max_ with the ratio of alkaline Pi to main Pi (Pi_2_/Pi); and (**c**) Q_max_ with the concentration of phosphodiesters ([PDE]). Further correlations of the ^31^P-MRS parameters measured at rest: (**d**) F_ATP_ with [PDE]; (**e**) Pi_2_/Pi with [PDE]; and (**f**) Pi-to-ATP exchange rate constant (k_ATP_) with Pi_2_/Pi. 95% confidence intervals are also depicted.

In addition, we have found correlations between the metabolic parameters extracted from the ^31^P-MRS measurements performed at rest and the oxidative metabolism markers measured in a dynamic exercise-recovery experiment. The [PDE] correlated negatively with Q_max_ (r = −0.51, p = 0.005), while both [Pi_2_] and Pi_2_/Pi correlated with Q_max_ positively (r = 0.68, p = 0.0001 and r = 0.65, p = 0.0002, respectively). Q_max_ significantly correlated also with the k_ATP_ (r = 0.51, p = 0.005) and F_ATP_ (r = 0.63, p = 0.0003). Several correlations were also found between the different parameters of muscular energy metabolism measured at rest. The [PDE] correlated negatively with [Pi_2_] (r = −0.63, p = 0.0003), Pi_2_/Pi (r = −0.63, p = 0.0003), k_ATP_ (r = −0.54, p = 0.003), and F_ATP_ (r = −0.59, p = 0.001). Both [Pi_2_] and Pi_2_/Pi were correlated with k_ATP_ (r = 0.41, p = 0.029 and r = 0.52, p = 0.005), as well as with F_ATP_ (r = 0.59, p = 0.001 and r = 0.45, p =  = 0.018). All correlations of the evaluated metabolic parameters with the [PDE] were also significant for the [GPC]. Representative correlations are depicted in Fig. 3.

Multivariate stepwise regression analysis of Q_max_ including physiological and metabolic parameters derived from ^31^P-MRS data acquired at rest, identified [Pi_2_] (r^2^ = 0.46, adjusted r^2^ = 0.44, p = 0.0001) as the strongest and F_ATP_ (r^2^ = 0.54, adjusted r^2^ = 0.50, p = 0.00001) as the second-strongest independent predictor of Q_max_. Detailed results are given in Table 2.

## Discussion

In this study, we compared parameters of skeletal muscle metabolism, measured by static and dynamic ^31^P-MRS methods, between a group of overweight-to-obese sedentary subjects, who are prone to diabetes, and a group of lean active individuals. We have found that the combination of increased BMI and sedentary lifestyle leads to significant differences in the alkaline Pi pool in skeletal muscle, as well as in other metabolic ^31^P-MRS parameters, such as the concentration of PDE, the Pi-to-ATP metabolic flux, and mitochondrial capacity. In addition, significant correlations were found between the concentration of PDE, the alkaline Pi_2_/Pi ratio, and the resting Pi-to-ATP exchange rate and flux, measured by ^31^P-MRS techniques at rest, and the maximal mitochondrial oxidative flux, measured by an exercise-recovery experiment.

Dynamic ^31^P-MRS provides a parameter closely related to training status, i.e., the mitochondrial capacity (Q_max_) of the muscle tissue^15^. This was also demonstrated in our study, as the Q_max_ of the overweight-to-obese sedentary subjects was significantly lower when compared to active, lean individuals. The correlation between Q_max_ and BMI found in this study can be explained by the decreased physical activity in more obese individuals, as our regression analysis showed a primary connection of Q_max_ with other parameters of muscle metabolism and not with BMI. This is in good agreement with a recent *in vitro* study, which found no differences in mitochondrial respiratory capacity and mitochondrial content in myocellular tissue samples between lean and obese subjects with similar training status^29^. Similarly, a different *in vivo* study did not find any changes in mitochondrial capacity in humans after weight reduction stimulated by diet-only; however, if combined with increased physical activity, an improvement in aerobic capacity was observed^30^.

Significantly higher myocellular PDE levels were found in our Ob/Sed subjects when compared to the L/Ac group. This was further supported with the positive correlation found between [PDE] and BMI. This is in good agreement with the finding of a previous report by Szendroedi *et al.*^26^ in subjects with a comparable physical activity index. A correlation of [PDE] and age was also reported, in the current study and in^26^,^31^; however, our regression analysis showed that physical activity and BMI, rather than age, primarily predict the PDE levels (data not shown). The increased spectral resolution of the 7 T MR system, used in our study, reveals that the measured [PDE] is mainly attributable to [GPC], with only a small contribution from glycero-phosphoethanolamine ([GPE]), and that, in fact, it is the [GPC] that is responsible for the differences between the two groups. This separation in PDE signals was not visible in the previous study performed at 3 T^26^.

The ratio of alkaline Pi_2_ to cytosolic Pi (Pi_2_/Pi) was lower in the Ob/Sed group in comparison to the L/Ac group. Recently, van Oorschot *et al.* reported a dependence of Pi_2_/Pi, measured in the vastus lateralis, on the training status, when they compared highly trained runners with normally active individuals^28^. The potential influence of BMI was, however, not considered in the aforementioned study. The results of our study suggest such a dependence of the Pi_2_/Pi ratio in skeletal muscle on BMI, as a linear correlation between BMI and Pi_2_/Pi was found. Nevertheless, the results of our regression analysis identified only [GPC] and Q_max_ as the primary predictors of the Pi_2_/Pi (data not shown). The differences in Pi_2_/Pi found between the groups can be directly attributed to the changes in [Pi_2_], which was also significantly higher in the L/Ac group in comparison to the Ob/Sed group.

Although the mean F_ATP_ values of our Ob/Sed group (F_ATP_ = 0.25 ± 0.06 mM.s^−1^) are still above the decreased values reported previously in patients with type 2 diabetes (F_ATP_ = 0.21 ± 0.05 mM.s^−1^)^25^ the physical inactivity together with the overweight of our volunteers caused a significant reduction in the myocellular Pi-to-ATP metabolic flux, when compared to L/Ac individuals (F_ATP_ = 0.31 ± 0.04 mM.s^−1^).

### Interrelations between metabolic parameters measured by dynamic and static ^31^P-MRS

We report several correlations between the parameters of static ^31^P-MR spectra, exchange rates and metabolite fluxes measured by ST at rest, and oxidative metabolism markers measured by exercise-recovery experiments. The alkaline Pi_2_ resonance is suspected to represent mitochondrial density in the muscle tissue and depends on the amount of regular physical activity^28^. Its relation to training status was also confirmed in our study, as the [Pi_2_], as well as Pi_2_/Pi, positively correlated with the maximal oxidative flux (Q_max_), determined during the dynamic experiment, and also with the k_ATP_ and F_ATP_, defining the Pi-to-ATP exchange rate and metabolic flux. Linear correlations between Q_max_ and metabolic parameters measured by ST experiments at rest (i.e., k_ATP_ and F_ATP_) reported in our previous study on overweight-to-obese subjects^22^, were also found in this study combining the two different population groups. In addition, the multivariate regression analysis identified [Pi_2_] and F_ATP_ as independent predictors of Q_max_, suggesting the potential use of highlyspectrally resolved static ^31^P-MRS at 7 T and ST as alternative techniques to dynamic exercise-recovery experiments.

The identified correlation between [PDE] and measured Pi-to-ATP metabolite flux (F_ATP_) is in good agreement with a recent report by Szendroedi *et al.*^26^. Significant correlations were found also between [PDE] and mitochondrial capacity (Q_max_), as well as other ^31^P-MR parameters of muscle metabolism measured at rest, i.e., [Pi_2_], Pi_2_/Pi, and k_ATP_. In addition, the main contributors to total [PDE] were analyzed and all [PDE] correlations were also significant for [GPC]. Our results suggest the potential of using [PDE], or, if distinguishable (i.e., at 7 T) directly, the [GPC], as asurrogate biomarker of skeletal muscle energy metabolism. Although it is not perfectly clear what links GPC to muscle energy metabolism, previous studies support this finding^32^,^33^,^34^,^35^,^36^,^37^. In particular, Farber *et al.*, studying a model of membrane defect of Alzheimer’s disease, reported that an inhibition of oxidative phosphorylation causes accumulation of GPC through accelerated PC turnover^34^. Impaired oxidative metabolism and elevated PDE levels have been also reported in patients with spinal cord injury^35^ and congenital lipodystrophy^36^. Muscular PDE content was also related to glucometabolic control in type II diabetes^26^. Furthermore, excessive amounts of PDE have been reported in fibromyalgia^33^, Duchenne muscular dystrophy^32^, or Becker muscular dystrophy^37^, connecting abnormal membrane metabolism with muscle dysfunction. Nonetheless, further investigations of this relation are still necessary.

The findings of this study support our previous report on correlations between dynamic and ST parameters in this Ob/Sed group^22^ and provide additional information through analysis of Pi_2_/Pi and GPC, and moreover, by comparison to a lean active group of individuals. As to the technical limitations of our study, we should note that although care was taken to perfectly reposition the subject in the second MR system, when applicable, some small mislocalizations could not be fully excluded. The effect of individual anatomy must be also considered, as the localization through the sensitivity of the surface coil used in this study might cover different portions of the quadriceps muscles between subjects. Localization techniques, recently proposed for dynamic examinations of the lower leg muscles, e.g., frequency selective ^31^P-MRI^38^,^39^,^40^, semi-LASER for single voxel localization^41^ or depth-resolved surface coil MRS^42^, could be used in future studies to measure muscle-specific metabolism. However, the muscles of the quadriceps covered by the sensitivity volume of the used surface coil are all active during knee-extension^43^, and, therefore, the inter-subject variability of the covered muscle volumes should have had only a minor effect on our results.

In conclusion, overweight-to-obese sedentary pre-diabetics exhibit increased concentrations of glycero-phosphocholine, a lower amount of alkaline Pi, a slower Pi-to-ATP exchange rate, and decreased mitochondrial capacity compared to lean active individuals. Associations found between the parameters of myocellular metabolism measured at rest and during exercise suggest that highly spectrally resolved static ^31^P-MRS and saturation transfer measurements at rest could provide markers of muscle mitochondrial metabolism.

## Methods

Fifteen young, overweight-to-obese, sedentary individuals (10/5 male/female; age 34.6 ± 7.1 years) with a body mass index (BMI) ≥ 27.0 kg.m^−2^, a sedentary lifestyle without regular physical activity, no pharmacotherapy, and no medical history of type 2 diabetes were recruited for this study and classified as the overweight-to-obese/sedentary (Ob/Sed) group. Thirteen of these volunteers had already participated in our previous study on the interrelations between mitochondrial capacity and Pi-to-ATP exchange rates in this particular type of population^22^. Fifteen young, lean, physically active participants (10/5 male/female; age 29.3 ± 5.5 years) were recruited for the current study as the control lean/active (L/Ac) group.

Written, informed consent was obtained from each participant in the study after an explanation of the purpose, nature and potential risks of the study. The examination protocol was approved by the appropriate institutional ethical boards of the Medical University of Vienna and of the University Hospital Bratislava, Comenius University Bratislava, and the study was carried out in accordance with the approved guidelines.

### Physiological tests

Within a week before the MR examination, the participants underwent a physical examination and physiological testing. BMI was measured by an analog weight scale and standard measuring tape. Bioelectric impedance, measured using an Omron BF511 (Omron Healthcare, Matsusaka, Japan), was used to evaluate total adiposity (%Fat) and to estimate the lean body mass (LBM). The maximal aerobic capacity (i.e., whole-body oxygen uptake [VO_2max_]) was measured during an incremental exercise test performed on a Lode Corival cycle ergometer (Lode, Groningen, The Nederlands). Continuous measurement of the gas exchange rate was obtained with the Ergostik (Geratherm Respiratory, Bad Kissingen, Germany), and the maximal oxygen consumption rate was expressed relative to LBM. The ergometry was performed at least three days prior to the MR examinations. The activity level was evaluated based on two working days and a weekend of accelerometer recordings and expressed as the number of steps per 24 hours.

### ^31^P-MRS

Each participant underwent the entire MR examination protocol in one day, starting two hours after a standardized meal. The dynamic ^31^P-MRS exercise-recovery experiment was performed on either a 7 T MR system (Magnetom, Siemens Healthcare, Erlangen, Germany) or a 3 T MR system (TIM Trio) from the same manufacturer, due to initial compatibility problems of our ergometer (Quadspect, Ergospect, Innsbruck, Austria) with the 7 T. Dual-tuned (^31^P-^1^H) circular surface coils (10 cm diameter, Rapid Biomedical, Rimpar, Germany), with similar sensitivity volumes^22^ were used on both MR systems. The use of two MR systems, equipped with the same ergometer and surface coils with a similar sensitivity volume, has recently been shown to have no effect on the metabolic data derived from dynamic ^31^P-MRS^44^.

Static ^31^P-MRS experiments were performed exclusively at 7 T, as the increased spectral resolution is necessary for separation of the Pi_2_, as well as the GPE and GPC signals^27^, and the increase in signal-to-noise ratio allows significant reduction in measurement time of the ST experiment, compared to 3 T^45^. The subjects were investigated while lying inside the MR scanner with the surface coil fixed to the quadriceps femoris muscle (Fig. 1a) and the coil positions were marked to allow precise repositioning in the other MR system, if applicable. When the dynamic measurements were performed at 3 T (i.e., in case of first 10 Ob/Sed subjects), the order of examinations was randomized to allow simultaneous examinations of two subjects; otherwise the measurements at rest were always performed prior to the exercise-recovery experiment.

For the assessment of intramyocellular metabolite concentrations and the Pi_2_/Pi ratio, a pulse-acquire ^31^P-MR spectrum (acquisition delay = 0.4 ms; repetition time = 15 s; bandwidth = 5 kHz; 16 averages in 4 minutes) was acquired at rest (Fig. 1b) and corrected for longitudinal relaxation times, as measured for ^31^P muscle metabolites at 7 T^27^,^46^. The γ-ATP signal was used as an internal concentration reference, assuming a stable ATP concentration of 8.2 mM in the skeletal muscle^12^.

The exchange rate between ATP and Pi (i.e., ATP synthesis) was investigated using an ST experiment applying continuous irradiation, and the apparent longitudinal relaxation time (T_1_^app^) was determined via an inversion recovery experiment, as described previously^45^. The total measurement time of the ST experiment was under 9 minutes.

The exercise-recovery protocol involved six minutes of repeated knee extensions against an air pressure, set to 30% of the maximal voluntary contraction force, once every repetition time (i.e., 2 s), followed by six minutes of recovery^15^. The volunteers were instructed by an audio signal to time the contraction-relaxation periods, so that the spectra were acquired always in the relaxed state of the muscle.

### Analyses and calculations

Due to patient noncompliance (n_Ob/Sed_ = 1) and technical problems (n_Ob/Sed_ = 1), 28 complete datasets and one incomplete dataset (dynamic ^31^P-MRS only), were available for analyses.

All acquired ^31^P-MR spectra were analyzed using jMRUI software with the AMARES time domain fitting algorithm^47^. The resonance lines of PCr, two Pi signals, and two PDEs—glycero-phosphocholine (GPC) and glycero-phosphoethanolamine (GPE)—were fitted as single Lorentzians, whereas γ- and α-ATP were fitted as doublets and β-ATP as a triplet. The line width of the Pi_2_ peak was constrained with respect to the line width of the main Pi peak, and the expected frequency difference between Pi_2_ and Pi was set to ~0.4 ppm, to ensure a good fit for the Pi_2_ peak^27^,^28^. The shift in resonance position between PCr and Pi signals in parts per million (δ) was used to calculate intramyocellular pH^48^, according to the Henderson-Hasselbalch equation: pH = 6.75 + log((δ−3.27)/(5.63−δ)). The free cytosolic ADP concentration ([ADP]) was calculated according to the method described by Kemp *et al.*^49^, assuming that 15% of total creatine [Cr] was not phosphorylated in the resting state^50^.

The chemical exchange rate constant (k_ATP_) was calculated from the fractional reduction of Pi magnetization upon selective saturation of γ-ATP (Fig. 1c)^45^. The resting unidirectional forward exchange flux was then calculated as F_ATP_ = k_ATP_ × [Pi].

To calculate the time constant of PCr resynthesis (τ_PCr_), the PCr signal changes during the recovery period of the dynamic experiment (Fig. 1d) were fitted to a monoexponential function using MATLAB (MathWorks, Nattick, MA, USA). The initial PCr recovery rate (V_PCr_), which roughly represents ATP turnover at the end of exercise, was determined and used to calculate the maximal rate of oxidative phosphorylation (Q_max_) according to the ADP-based model of Michaelis and Menten^49^.

Data are presented as means ± standard deviations and compared between the groups by an unpaired Student t-test. The relationships between metabolic parameters were analyzed by linear regression analysis, using Pearson’s correlation coefficient, to estimate the strength of the relationship. Multivariate stepwise regression analysis for the dependent variable Q_max_ was performed using the independent variables (i.e., BMI, age, [PDE], [GPC], [Pi_2_], Pi_2_/Pi, k_ATP_, and F_ATP_). A similar multivariate regression analysis was performed for the dependent variables [PDE] and Pi_2_/Pi. The results were considered statistically significant at p < 0.05.

### Data. 

Parts of the data were presented as abstracts at the following meetings: ISMRM 2012 Melbourne Australia, ISMRM 2015 Toronto Canada. Some ^31^P-MRS data of the dynamic and ST experiment, from the Ob/Sed group exclusively, were published in the *NMR in Biomedicine* journal (ref. 22).

## Additional Information

**How to cite this article**: Valkovič, L. *et al.* Skeletal muscle alkaline Pi pool is decreased in overweight-to-obese sedentary subjects and relates to mitochondrial capacity and phosphodiester content. *Sci. Rep.* **6**, 20087; doi: 10.1038/srep20087 (2016).

## Figures and Tables

**Table 1:** Characteristics of the studied groups and results of muscle energy metabolism measurements via static ^31^P-MRS, saturation transfer, and dynamic experiments.

Variable	Overweight Obese/Sedentary	Lean/Active
N (female)	14 (5)°	15 (5)
Age (years)	34.6 ± 7.1	29.3 ± 5.5
BMI (kg.m^−2^)	30.4 ± 2.3	23.1 ± 2.6^*^
Body fat (%)	35.2 ± 7.1	18.3 ± 6.1^*^
LBM (kg)	62.4 ± 10.9	63.0 ± 15.6
VO_2max_ (mL.min^−1^.kg^−1^)	36.8 ± 5.3	45.9 ± 3.1^*^
Steps per 24 hours	6052 ± 1166	11093 ± 4074^*^
**static MRS**		
[PDE] (mM)	4.21 ± 1.12	2.82 ± 1.00^*^
[GPC] (mM)	3.95 ± 1.04	2.47 ± 0.98^*^
[GPE] (mM)	0.26 ± 0.27	0.23 ± 0.17
[Pi_2_] (mM)	0.18 ± 0.07	0.28 ± 0.06^*^
Pi_2_/Pi	0.05 ± 0.02	0.08 ± 0.02^*^
pH_rest_	7.06 ± 0.04	7.05 ± 0.03
[ADP]_rest_(μM)	10.1 ± 0.9	9.8 ± 0.6
**ST**		
k_ATP_ (s^−1^)	0.07 ± 0.02	0.08 ± 0.01
F_ATP_ (mM.s^−1^)	0.25 ± 0.06	0.31 ± 0.04^†^
k_CK_ (s^−1^)	0.27 ± 0.05	0.25 ± 0.05
F_CK_ (mM.s^−1^)	9.26 ± 2.36	8.66 ± 2.40
**Dynamic**		
PCr drop (% signal)	38.4 ± 19.4	40.4 ± 13.9
τ_PCr_ (s)	40.9 ± 14.0	42.6 ± 15.8
V_PCr_ (mM.s^−1^)	0.29 ± 0.10	0.32 ± 0.07
Q_max_ (mM.s^−1^)	0.50 ± 0.08	0.58 ± 0.07^†^
pH_end_exercise_	6.97 ± 0.14	6.90 ± 0.16
[ADP]_end exercise_(μM)	47.8 ± 32.0	40.9 ± 15.5

Data are given as mean ± standard deviation. °For one volunteer from the overweight-to-obese sedentary group, only dynamic experiment data are available. Significant differences (unpaired t-test) between the groups are depicted as follows: ^*^p < 0.01; ^†^*p* < 0.05.

**Table 2:** Results of multivariable stepwise regression of Q_max_ (dependent variable) and physiological and metabolic variables measured at rest (independent variable).

Independent variables per Q_max_	*F* value	*p* value
Pi_2_^*^	22.035	0.0001
F_ATP_^*^	14.653	0.0001
Pi_2_/Pi	<0.05	0.217
BMI	<0.05	0.536
k_ATP_	<0.05	0.537
GPC	<0.05	0.689
PDE	<0.05	0.940
age	<0.05	0.961

^*^Variables accepted into the model as predictors; all other variables not accepted into the model.
